# The Beneficial Role of *Lactobacillus paracasei* subsp. *paracasei* NTU 101 in the Prevention of Atopic Dermatitis

**DOI:** 10.3390/cimb46030143

**Published:** 2024-03-09

**Authors:** Chin-Feng Liu, Tsung-Wei Shih, Chun-Lin Lee, Tzu-Ming Pan

**Affiliations:** 1 Continuing Education Program of Food Biotechnology Applications, National Taitung University, 684, Sec. 1, Zhonghua Rd.,Taitung 950017, Taiwan; cfliu@nttu.edu.tw; 2 SunWay Biotech Co., Taipei 10617, Taiwan; tw.shih@sunway.cc; 3 Department of Life Science, National Taitung University, 369, Sec. 2, University Rd., Taitung 95092, Taiwan; 4 Department of Biochemical Science and Technology, National Taiwan University, Taipei 10617, Taiwan

**Keywords:** atopic dermatitis, NTU 101, allergic reaction

## Abstract

Atopic dermatitis (AD) is a recurrent allergic disease characterized by symptoms such as itching, redness, swelling, dryness, scaling skin, inflammation, and tissue damage. The underlying pathogenesis of AD remains unclear. Steroid drugs are commonly used in the clinical treatment of AD; however, their long-term use may lead to associated complications. Numerous studies have indicated that probiotics could modulate the immune system, enhance immune function, or suppress excessive immune responses. In this study, *Lactobacillus paracasei* subsp. *paracasei* NTU 101 (NTU 101) was orally administered for a duration of 4 weeks, followed by the induction of AD using ovalbumin (OVA) in a mouse model. The skin condition of the stimulated site was observed during the induction period. Subsequently, the serum immunoglobulin E (IgE) content, splenocyte T cell typing, and skin histological interpretation were examined to evaluate the efficacy of NTU 101 in alleviating AD symptoms in allergen-exposed animals. The findings indicated that administering NTU 101 beforehand effectively alleviated skin symptoms in animals with AD. It reduced the infiltration of inflammatory cells in skin tissue sections, and compared to the OVA group, there was a significant reduction in the thickening of the epidermal cell layer (decreased from 89.0 ± 20.2 µM to 48.6 ± 16.0 µM) and dermis layer (decreased from 310.3 ± 69.0 µM to 209.7 ± 55.5 µM). Moreover, the proportion of regulatory T (Treg) cells and T helper 2 (Th2) cells in splenocytes significantly increased, while the proportions of T helper 1 (Th1) and T helper 17 (Th17) cells did not differ. It is speculated that the potential mechanism by which NTU 101 prevents AD involves increasing the expression of Forkhead box protein P3 (FOXP3) and promoting Treg cell maturation, thereby alleviating allergic reaction symptoms associated with AD.

## 1. Introduction

Atopic dermatitis (AD) is a chronic inflammatory skin condition characterized by pruritus (itchy skin). The etiology of AD involves a complex interplay between genetic and environmental factors [1]. The severity of AD varies based on race, age, and gender [2]. Industrialized countries have witnessed a rising incidence and prevalence of AD, resulting in a significant impact on quality of life and economic burden [3].

The treatment of AD typically requires long-term patient cooperation and can be classified as topical or systemic. In cases of bacterial infection, concurrent administration of antibiotics is necessary to prevent complications [4]. However, oral corticosteroids, while providing rapid symptom relief for some patients with acute AD, are associated with serious side effects. In cases of severe relapsing AD, allergen testing, including immunoglobulin E (IgE) evaluation, is often conducted to identify specific allergens and prevent disease recurrence [5].

Recent advancements in DNA sequencing technology have shed light on the role of the gut microbiota in physiological and pathological processes, including immune responses, metabolism, and disease development. Gut dysbiosis has been closely linked to various clinical conditions. In addition to the lymphatic system, immune barriers in the skin, upper respiratory tract, lungs, and gut play critical roles in protecting the body against foreign pathogens. Among these barriers, the intestinal mucosa is particularly robust, accounting for over 70% of the overall immune function. The “gut–skin” axis has emerged as a concept that highlights the interplay between the gut and skin in the prevention and treatment of AD. The gut and skin share similarities and are integral parts of the immune and endocrine systems [6]. The occurrence of intestinal diseases is often accompanied by cutaneous manifestations, suggesting a potential bidirectional relationship [7]. Consequently, targeting alterations in the gut microbiome may offer an alternative approach to modulating immune responses and improving skin health in AD patients.

Probiotics and prebiotics are widely recognized for their significant impact on gut health. Probiotics are live microorganisms that, when administered in adequate amounts, confer health benefits on the host. They help restore and maintain a healthy balance of gut microbiota, promoting a favorable environment for overall gut health [8]. These beneficial microorganisms can modulate immune responses, enhance intestinal barrier function, produce antimicrobial substances, and influence the metabolism of dietary components [9]. Probiotics have also been recognized for their potential benefits in promoting skin health. Several studies have explored the use of probiotics in managing various dermatological conditions, including AD, acne, rosacea, and wound healing [10,11]. The skin and gut have a bidirectional communication pathway known as the “gut–skin axis”. The gut microbiota can influence the skin microbiota and modulate immune responses, inflammation, and barrier function, which are crucial factors in maintaining skin health [12]. By improving gut health and modulating the gut microbiota composition, probiotics may indirectly impact skin health. Probiotics can exert their beneficial effects on the skin through various mechanisms. They can enhance skin barrier function, regulate the immune response, and exhibit antimicrobial and anti-inflammatory properties [13]. Furthermore, probiotics can produce bioactive compounds, such as antimicrobial peptides and short-chain fatty acids, which contribute to skin health [14]. While the precise mechanisms of probiotic action on the skin are still being elucidated, numerous clinical studies have demonstrated promising results. For instance, the supplementation of specific probiotic strains has been shown to decrease the severity and symptoms of atopic dermatitis (AD), enhance acne lesions, and alleviate symptoms of rosacea [11], exhibiting favorable effects in the prevention and treatment of chronic inflammatory skin diseases [15].

NTU 101 was originally isolated from healthy infants [16]. In previous studies, this strain has shown promising results in immune modulation and reducing inflammation in the host [17,18]. In this study, NTU 101 will be administered as a probiotic prior to inducing an animal model of AD to assess its potential for preventing and alleviating allergic symptoms in AD.

## 2. Materials and Methods

### 2.1. Sample Preparation

*Lactobacillus paracasei* subsp. *paracasei* NTU 101 (NTU 101) in powder form, containing 1 × 10^11^ CFU/g, was obtained from SunWay Biotech Co., Ltd. (Taipei, Taiwan). *Lactobacillus rhamnosus* ATCC 53103 was purchased from the Bioresource Collection and Research Center (Hsinchu, Taiwan). It was made into lyophilized powder, and the viable count was maintained at 1 × 10^11^ CFU/g before use. Prednisolone was purchased from Sigma-Aldrich (St. Louis, MO, USA), a synthetic corticosteroid primarily used to treat severe allergic disease. Ovalbumin (OVA) is a T cell-dependent antigen commonly utilized as a model protein for studying antigen-specific immune responses in mice. The preparation method of OVA is divided into two ways. (1) Systemic sensitization by intraperitoneal injection of OVA. The adjuvant used per animal is 2 mg Al (OH)_3_ dissolved in 0.2 mL 0.9% sterile saline in an autoclave and left at room temperature for at least 30 min. Twenty μg of OVA was dissolved in 0.2 mL of adjuvant and administered to animals by intraperitoneal injection; (2) OVA localized skin irritation and sensitization. Dissolve 100 µg OVA in 100 µL 0.9% saline to locally irritate the animal’s skin [19,20].

### 2.2. Animal Grouping and Experiment Schedule

**Figure 1:**
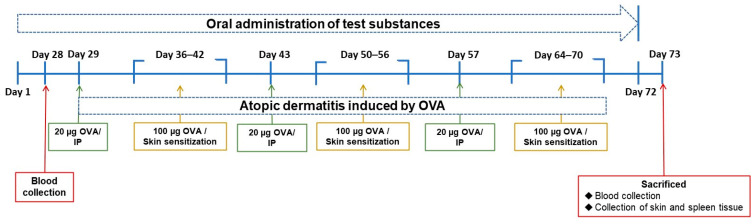
Experimental schedule in this study.

Balb/c mice at 5 weeks of age were purchased from the BioLasco Co. (Taipei, Taiwan). The mice were randomized and housed in stainless steel cages in an OVAled environment with a relative humidity of 50 ± 10%, room temperature of 23 ± 2 °C, and a 12-h light-dark cycle. According to the “Animal and Raw Material Management Procedure” (ATRI-ATL-QP-008) method, place an animal label card outside each breeding cage, indicating the IACUC number (No. 106038), animal strain, sex, group, animal number, date of birth, test cycle, and processing method. In this experiment, the average body weight of the animals prior to testing the substance was 16.63 ± 0.10 g. The error between each experimental group did not exceed 20%. Forty mice were randomly divided into five groups. The control group (NOR group) was inducted with saline and had a normal diet. In the OVA group, systemic sensitization and local irritation of the skin surface were induced by OVA, and they had a normal diet. In the clinical medication group (CM group), systemic sensitization and local irritation of the skin surface were induced with OVA after 28 days of prednisolone pre-feeding and a normal diet. In the NTU 101 group, systemic sensitization and local irritation of the skin surface were induced with OVA after 28 days of NTU 101 (1 × 10^11^ CFU/day) pre-feeding and a normal diet. In the LGG group, systemic sensitization and local irritation of the skin surface were induced with OVA after 28 days of *L. rhamnosus* GG ATCC 53103 (1 × 10^11^ CFU/day) pre-feeding and a normal diet. For food intake, the amount and remaining amount of food provided to the experimental mice on a weekly basis were recorded. In the experimental OVA induction phase, systemic sensitization was initiated through intraperitoneal injection of OVA (20 μg/200 μL/mouse) over the course of 1 week. Subsequently, the skin was stimulated with OVA (100 μg/100 μL/mouse) for a duration of 7 days. This cyclic process was reiterated three times (Figure 1). This experiment simulates human administration, and all the tested substances are administered by intragastric administration for 72 consecutive days.

### 2.3. AD Score

After stimulating the skin with OVA, observe the skin condition, score, and take pictures every 3–4 days. The scoring method is calculated as follows: Asymptomatic: 0 points; Scratches: 1 point; Redness: 2 points; Skin breakage/desquamation: 3 points; Mucus, body fluid production: 4 points; Mucus, fluid production/pus/blood: 5 points.

### 2.4. Blood Collection and Analysis

During the experiment, blood was collected in blood collection tubes without anticoagulant by orbital blood collection. After sacrifice, blood was collected by the heart in blood collection tubes with or without anticoagulant. Centrifuge the anticoagulant-free blood at 3500 rpm for 10 min at 4 °C and store the supernatant at −40 °C until use. For hematology, whole blood was mixed with EDTA, and a five-category analysis of the white blood cell count (neutrophils, lymphocytes, monocytes, eosinophils, and basophils) was performed by using a hematology analyzer (ProCyte Dx, IDEXX Laboratories, Westbrook, MA, USA) and the applicable operating procedures (ATRI-ATL-ISOP-027).

### 2.5. Enzyme-Linked Immunosorbent Assay (ELISA)

This assay was used to measure the total serum IgE level before and after the test and the serum OVA-specific IgE level after the test. Total IgE (Cat. No. BTLE99-115, Bethyl) and OVA-specific IgE (Cat. No. BRAMCA2475KZZ, Bio-Rad, Hercules, CA, USA) levels in the serum were determined using commercially available kits, and the measurement methods conformed to the commercial kit operation manual.

### 2.6. Cell Clustering and Cytokine Detection

Splenocytes were isolated and stained with propidium iodide (PI), and flow cytometric analysis was performed to determine CD4^+^/IL-4^+^, CD4^+^/IL-17^+^, CD4^+^/IFN-γ^+^, and CD4^+^/CD25^+^/FOXP3^+^ expression by immune cells. Splenocyte isolation method: The spleen was cut into pieces using sterile scissors, ground using a cell strainer (Bock) with a hole diameter of 70 μm and a 10 mL syringe plunger, suspended in 15 mL RPMI 1640 medium (4 °C), centrifuged at 4 °C for 10 min at 1000 rpm, and washed with PBS twice. After washing, the cell pellets were suspended in ammonium–chloride–potassium (ACK) lysis buffer (Becton Dickinson, San Diego, CA, USA) at room temperature and uniformly mixed for 10 min. For preparing a pure splenocyte-containing suspension, the mixture was then centrifuged at 4 °C at 1000 rpm for 10 min and washed with 1× PBS (4 °C) to remove red blood cells. Propidium iodide (PI) staining was performed as follows: 1 mL of fresh splenocytes were stained with 10 μL PI (50 μg/mL) for 10 min in the dark, and the presence of CD4^+^/IL-4^+^, CD4^+^/IL-17^+^, CD4^+^/IFN-γ^+^, and CD4^+^/CD25^+^/FOXP3^+^ was detected using a computer-based flow cytometer and analyzed using the WinMDI 2.9 Software (Scripps Research Institute, La Jolla, CA, USA).

### 2.7. Detection of Immune Cell Populations Using Monoclonal Antibodies and Flow Cytometry

The splenocytes were suspended in a fluorescence-activated cell sorting buffer (BD Biosciences, Franklin Lakes, NJ, USA). The BD Cytofix/Cytoperm™ Kit (Cat. No. 554714, BD) was used for fixing and cell membrane perforation, and paired staining was performed using the following monoclonal antibodies: FITC anti-mouse CD4 monoclonal antibody (clone: RM4-5, eBioscience, San Diego, CA, USA); PE rat anti-mouse CD25 monoclonal antibody (clone: PC61.5, eBioscience); PerCP-Cy™ 5.5 rat anti-mouse IFN-γ monoclonal antibody (clone: XMG1.2, BD); PerCP-Cy™ rat anti-mouse IL-4 monoclonal antibody (clone: 11B11, BioLegend, San Diego, CA, USA); PerCP-Cy™ 5.5 rat anti-mouse FOXP3 monoclonal antibody (clone: FJK-16s, Invitrogen, Carlsbad, CA, USA); PerCP-Cy™ 5.5 rat anti-mouse/rat IL-17A monoclonal antibody (clone: eBio17B7, eBioscience). A flow cytometer was used to analyze the percentage of fluorescence intensity in the specimens, and the WinMDI 2.9 Software was used for analysis. CD4^+^ was expressed by T-helper (Th) cells; CD4^+^/IFN-γ^+^ by Th1 cells; CD4^+^/IL-4^+^ by Th2 cells; CD4^+^/IL-17^+^ by Th17 cells; and CD4^+^/CD25^+^/FOXP3^+^ by T-regulatory (Treg) cells.

### 2.8. Histopathological Analysis

The skin tissue at the irritation site was surgically resected and fixed with a 10% neutral formalin solution for histopathological analysis. The skin tissue was cut transversely and placed in the embedding box, and paraffin tissue blocks were prepared by subjecting the tissue to dehydration, infiltration with paraffin, and embedding. Then, the tissue blocks were cut using a paraffin tissue microtome (Leica RM 2145, Nussloch, Germany) into 4 μm sections, stained with hematoxylin and eosin (H&E) stain, and subjected to immunohistochemical analysis for determining the antigen quantity of thymic stromal lymphopoietin (TSLP) (Cat. No. PA5-20320, Thermo, Waltham, MA, USA). Thereafter, the severity of OVA-induced AD was determined by observation under a light microscope [21].

### 2.9. Statistical Analysis

Experimental results were expressed as the mean ± standard deviation. All data were analyzed by performing Dunnett’s *t*-test or Scheffe’s test using a one-way ANOVA of the SPSS system. TSLP antigen expression was analyzed by performing the Mann–Whitney U-test. Differences between groups were considered statistically significant if the *p*-value was less than 0.05; * denotes *p*-value less than 0.05 (*p* < 0.05); ** denotes *p*-value less than 0.01 (*p* < 0.01); *** denotes *p*-value less than 0.001 (*p* < 0.001).

## 3. Results

### 3.1. Changes in Body Weight and Food Intake

At the beginning of the experiment (Day 0), as shown in Table 1, the animals’ body weights in each group were similar. During the early stage of the test induction (Days 0–28), the animals’ weight increased steadily, with the CM group’s body weight significantly lower than that of the OVA group on Days 14 and 28 (*p* < 0.05; *p* < 0.01), while there was no significant difference in the other groups. During the skin irritation induction period (Days 42, 56, and 70), the average body weight of each group decreased. This was presumed to be due to the need to bandage the animals for seven days during the skin irritation period to increase the contact time between OVA and the skin, resulting in decreased food intake and skin irritation. However, the animals recovered after the dressings were removed. During the skin irritation induction period, the experimental group’s animals maintained the highest body weight among the groups, and it was significantly higher than that of the OVA group on Days 42–70 (*p* < 0.05; *p* < 0.01). The body weight of the animals in the clinical treatment group was lighter than that of the other groups, and it was significantly lower than the OVA group on Days 35–73 (*p* < 0.05; *p* < 0.01; *p* < 0.001). Both the LGG and OVA groups were similar, but at the end of the experiment (Day 73), the animals’ body weight was lower than that of the OVA group (*p* < 0.05).

During the early induction period (Weeks 1–4), there was no statistical difference in food intake between each group. The systemic sensitization did not affect food intake during the induction period (Weeks 5–10), but during the skin irritation period (Weeks 8 and 10), the food intake was slightly reduced, presumably due to the bandaging and fixing required for the stimulation induction, which affected the animals’ appetite. However, the food intake of animals in each group was similar during the test period, and there was no statistically significant difference (Table 2).

### 3.2. Prophylactic Improvement of Skin Condition in AD with Probiotics

**Figure 2:**
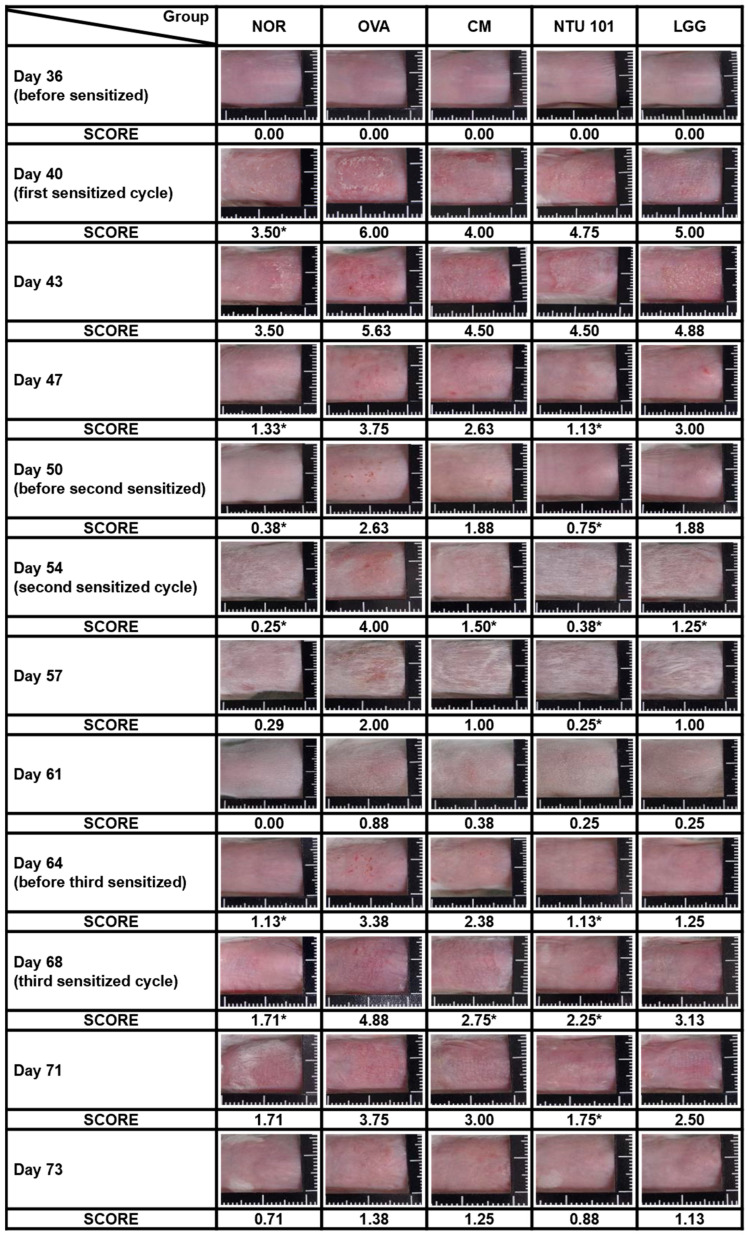
Representative images and clinical skin severity scores of sensitized skin sites in each group during the experimental period. NOR group was inducted with saline and had a normal diet; OVA group, systemic sensitization and local irritation of the skin surface were induced by OVA, had a normal diet; CM group, systemic sensitization and local irritation of the skin surface were induced with OVA after 28 days of prednisolone pre-feeding, had a normal diet. In the NTU 101 group, systemic sensitization and local irritation of the skin surface were induced with OVA after 28 days of NTU 101 (1 × 10^11^ CFU/day) pre-feeding and a normal diet. In the LGG group, systemic sensitization and local irritation of the skin surface were induced with OVA after 28 days of *L. rhamnosus* GG ATCC 53103 (1 × 10^11^ CFU/day) pre-feeding and a normal diet. The significance is compared with the OVA group (*: *p* < 0.05).

During the induction period, skin observations and recordings were taken once every 3 to 4 days, with three total skin stimulations. As shown in Figure 2, during the first skin stimulation period (Day 40), the average SCORE score of the stimulated skin in the OVA group was 6.00, which was significantly higher than the average score of 3.50 in the NOR animal group (*p* < 0.05). The average SCORE scores of the stimulated skin in the CM group were 4.00, in the NTU 101 group were 4.75, and in the LGG group were 5.00. Although lower than the OVA group, the differences were not statistically significant. When the animal was not in contact with the allergen (OVA), the skin condition of the animals in each group gradually returned to normal. On Days 47 and 50, the average SCORE scores of the stimulated skin in the NOR group were 1.13 and 0.38, respectively. In contrast, the OVA group had scores of 3.75 and 2.63, the CM group had scores of 2.63 and 1.88, the NTU 101 group had scores of 1.13 and 0.75, and the LGG group had scores of 3.00 and 1.88. These results showed that the OVA group had significantly higher scores than the NOR group (*p* < 0.001), and the NTU 101 group had significantly lower scores than the OVA group (*p* < 0.001) and were similar to the NOR group.

During the second skin stimulation induction with OVA (Days 54 and 57), it was found that the severity of dermatitis in the OVA group was slower than that of the first stimulation induction, and the recovery time was shortened. The average SCORE scores were 0.25 and 0.29 for the NOR group, 4.00 and 2.00 for the OVA group, 1.50 and 1.00 for the CM group, 0.38 and 0.25 for the NTU 101 group, and 1.25 and 1.00 for the LGG group. The statistical difference between the OVA group and the other groups was *p* < 0.01, while the difference between the CM group and the LGG group was *p* < 0.05. During the non-stimulation induction period (Days 61 and 64), the average scores of skin SCORE scores at the stimulated sites in each group were as follows: the NOR group was 0.00 and 1.13, the OVA group was 0.88 and 3.38, the CM group was 0.38 and 2.38, the NTU 101 group was 0.25 and 1.13, and the LGG group was 0.25 and 1.25. The results showed that the average score of the OVA group was higher, and the speed of skin recovery was slower than that of the other groups. After the animals were shaved on Day 64, the average score of the NTU 101 group was significantly lower than that of the OVA group (*p* < 0.05) and similar to the NOR group, but there was no statistical difference between the CM group, the LGG group, and the OVA group. During the third skin stimulation period (Days 68 and 71), the average SCORE scores of the stimulated skin in the NOR group were 1.71 and 1.71, respectively. In contrast, the OVA group had scores of 4.88 and 3.75, the CM group scored 2.75 and 3.00, the NTU 101 group showed scores of 2.25 and 1.75, and the LGG group had scores of 3.13 and 2.50. Upon comparing these values with those of the OVA group, the average SCORE of the NTU 101 group and CM group were found to be significantly lower (*p* < 0.01; *p* < 0.05) on Day 68, while there was no statistically significant difference compared to the LGG group. At the end of Day 73, when the animals were sacrificed, the average SCORE of the irritation site was as follows: NOR group, 0.71; OVA group, 1.38; CM group, 1.25; NTU 101 group, 0.88; and LGG group, 1.13. Although the skin recovery rate in the NTU 101 group was higher than that in the other treatment groups and similar to that in the NOR group, the difference was not statistically significant. These results indicate that oral pre-administration of NTU 101 in the AD-induced BALB/c mouse model contributed to restoring skin normalcy, thereby reducing the SCOREs for AD.

### 3.3. Preventive Effect of Probiotics on AD through Histopathological Analysis

**Figure 3:**
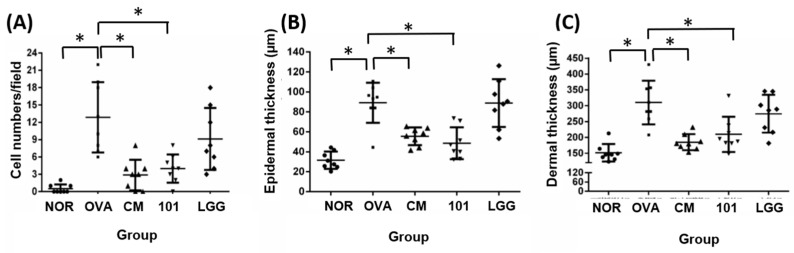
Histological changes in inflammatory cell infiltration (**A**), epidermal thickness (**B**), and dermal thickness (**C**) in each group. H&E staining at 100× magnification and 500 micrometer (μm) ruler length. Each value was expressed as the mean ± SD (*n* = 8). Statistical comparison of differences between each group and the OVA group (*: *p* < 0.05).

**Figure 4:**
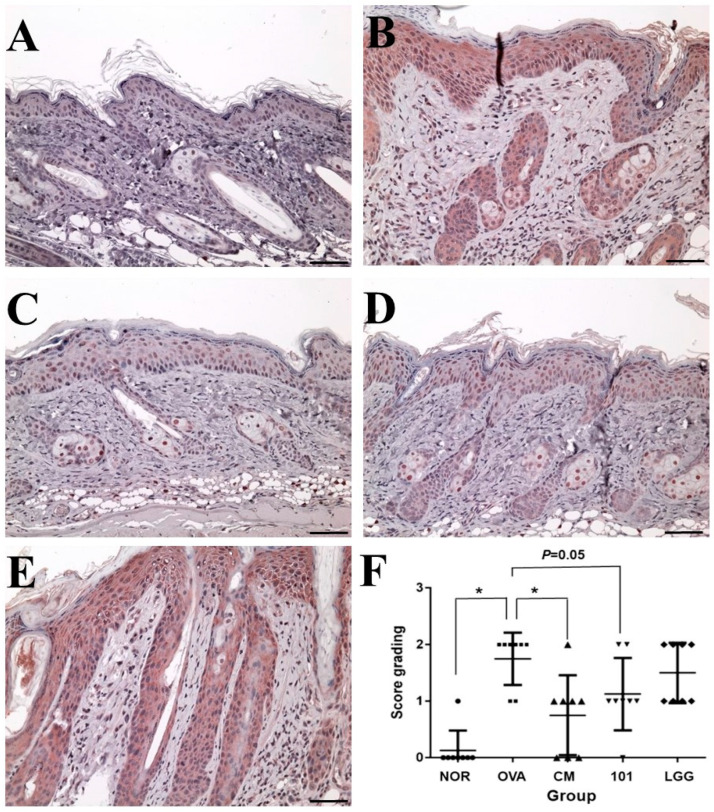
Changes in the expression amount of TSLP antigen in the epidermal cell layer of each group. (**A**): NOR group; (**B**): OVA group; (**C**): CM group; (**D**): 101 groups; (**E**): LGG group; (**F**): Scoring results for each group. Immunohistochemistry staining DAB color method, magnification 100 times, 500 micrometer (μm) ruler length. Each value was expressed as the mean ± SD (*n* = 8). Statistical comparison of differences between each group and the OVA group (*: *p* < 0.05).

The experiment aimed to evaluate the efficacy of NTU 101 in treating AD in animal models induced by OVA. After the animals were sacrificed, the skin of the stimulated part was taken for histological analysis. The indicators analyzed included inflammatory cell infiltration, epidermal cell layer thickness, dermis layer thickness (Figure 3), and TSLP antigen expression (Figure 4).

The results showed that the number of inflammatory cells (mainly eosinophils) in the lesion area was significantly lower in the NOR group (0 ± 0) compared to the OVA group (13 ± 6) (Figure 3A) (*p* < 0.01). The NTU 101 group (4 ± 2) and CM group (3 ± 3) also had significantly lower numbers of inflammatory cells compared to the OVA group (*p* < 0.01). The average thickness of the epidermal cell layer of the NOR group (31.5 ± 8.6 µM), the NTU 101 group (48.6 ± 16.0 µM), and the CM group (55.5 ± 8.9 µM) was smaller and statistically significant (*p* < 0.01) compared to the OVA group (89.0 ± 20.2 µM), while there was no statistical difference in the average thickness of the LGG group (88.8 ± 23.9 µM) (Figure 3B). The average thickness of the dermis in the lesion area of the OVA group (310.3 ± 69.0 µM) was significantly higher than that of the NOR group (151.3 ± 8.6 µM) (*p* < 0.01), while the average thickness of the NTU 101 group (209.7 ± 55.5 µM) and the CM group (185.0 ± 25.3 µM) was significantly smaller than that of the OVA group (Figure 3C) (*p* < 0.01).

TSLP antigen expression analysis showed that the expression level of TSLP antigen in the OVA group was significantly higher than that in the NOR group and the CM group (*p* < 0.01) (Figure 4), while the expression level of TSLP antigen in the NTU 101 group was slightly lower than that in the OVA group (*p* = 0.05). The total IgE content in the serum of the OVA group, NTU 101 group, and LGG group was significantly lower than that of the NOR group after four consecutive weeks of oral administration (*p* < 0.05). After induction with OVA stimulation, the total IgE content in the serum of each group increased, and the OVA group was significantly higher than the NOR group (*p* < 0.001). The CM group was significantly lower than the OVA group (*p* < 0.001). After induction with OVA, the content of specific IgE (OVA) in the serum of the OVA group, NTU 101 group, and LGG group all increased, and the OVA group was significantly higher than the NOR group (*p* < 0.001). The content of specific IgE (OVA) in the serum of the experimental group and the LGG group had no significant difference. Overall, the results suggest that oral administration of NTU 101 can help slow down evaluation indicators such as inflammatory cell infiltration, epidermal cell layer thickness, and dermis layer thickness in animal models of OVA-induced AD.

### 3.4. Changes in Specific IgE (OVA) Levels in Serum

**Figure 5:**
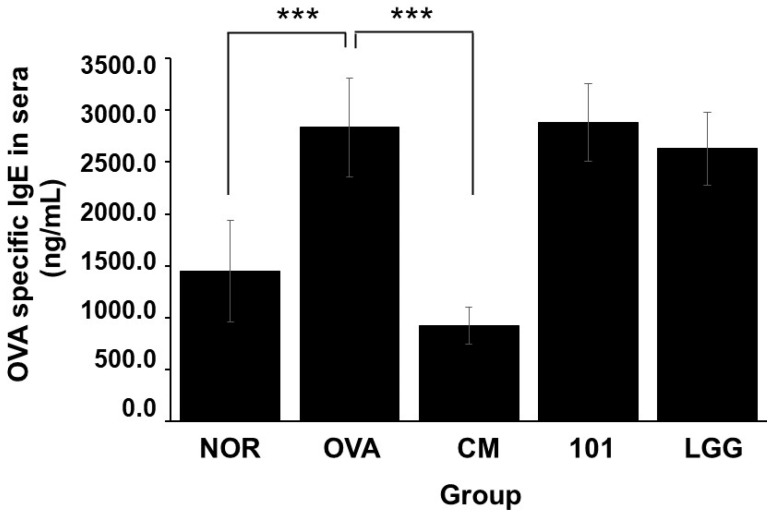
Changes in OVA-specific IgE in the serum of each group. Each value was expressed as the mean ± SD (*n* = 8). Statistical comparison of differences between each group and the OVA group (***: *p* < 0.001).

As shown in Figure 5, after inducing AD animal models using OVA, the serum content of specific IgE (OVA) increased in the OVA group, NTU 101 group, and LGG group. Notably, the NOR group exhibited significantly higher levels compared to the group of animals without abnormalities (*p* < 0.001). However, there were no significant differences in the serum-specific IgE (OVA) content between the NTU 101 group, LGG group, and NOR group.

### 3.5. Cell Classification and Cytokine Detection

**Figure 6:**
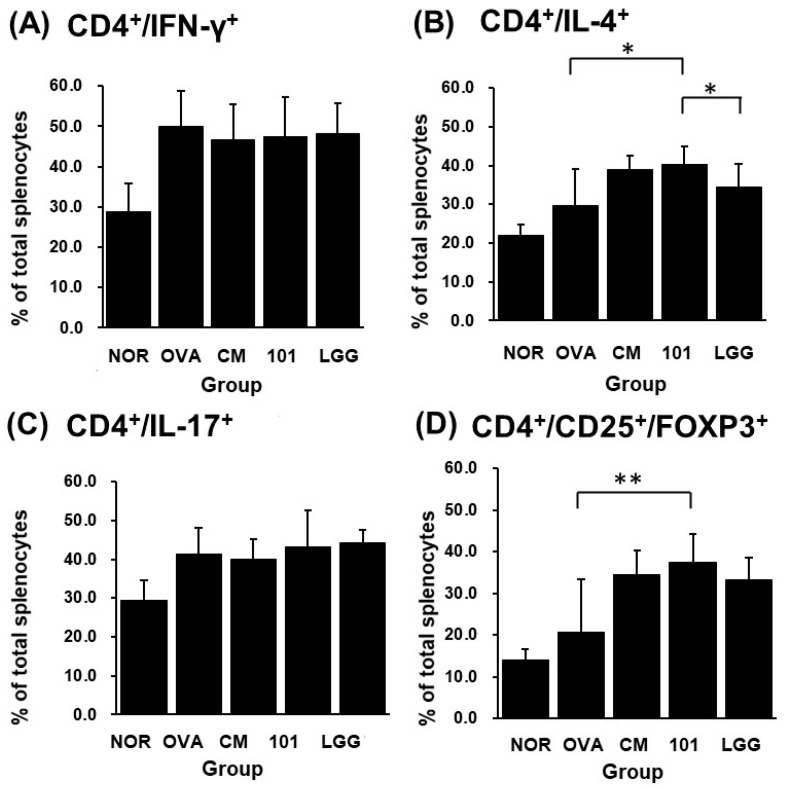
Effects of *Lactobacillus paracasei* subsp. *paracasei* NTU 101 on CD4^+^/IFN-γ^+^ (**A**), CD4^+^/IL-4^+^ (**B**), CD4^+^/IL-17^+^ (**C**), and CD4^+^/CD25^+^/FOXP3^+^ (**D**) in splenocytes from EC-sensitized mice. Each value was expressed as the mean ± SD (*n* = 8). Statistical comparison of differences between each group and the OVA group (*: *p* < 0.05; **: *p* < 0.01).

After sacrificing the animals at the end of the experiment, spleen tissue was removed for cell separation and staining, and PI, CD4^+^/IL-4^+^, CD4^+^/IL-17^+^, CD4^+^/IFN-γ^+^, CD4^+^/CD25^+^/FOXP3^+^ ratios were analyzed by flow cytometry. Regarding the death ratio of spleen cells in each group, the NTU 101 group had the lowest death ratio of spleen cells, and statistical results showed that the death degree of spleen cells in the NTU 101 group was significantly lower than that of the OVA group, CM group, and LGG group (*p* < 0.001). In terms of the proportion of immune cell populations in spleen cells of each group, the CD4^+^/IL-4^+^ population proportion in spleen cells of the NTU 101 group was significantly higher than that of the OVA group and the LGG group (*p* < 0.05) (Figure 6B). The population proportion of CD4^+^/IL-17^+^ and CD4^+^/IFN-γ^+^ in spleen cells showed no significant difference among the groups (Figure 6A,C). The population ratio of CD4^+^/CD25^+^/FOXP3^+^ in spleen cells showed that the NTU 101 group had a significantly higher ratio than the OVA group (*p* < 0.01), but there was no significant difference between the CM group and the LGG group (Figure 6D).

## 4. Discussion

To date, numerous reviews and meta-analyses have affirmed the effectiveness of probiotics in the clinical prevention and treatment of atopic dermatitis (AD). However, not all strains of lactobacilli prove effective in addressing AD. Typically, additional validation and screening are necessary to identify the most efficacious lactobacillus strains, taking into account their mechanisms of action against AD, optimal dosage, and the most favorable administration timing [22,23]. This research aims to explore the potential preventive effectiveness of the test substance NTU 101 against AD. The underlying mechanisms behind AD remain unclear; however, it is widely believed that environmental factors play a significant role in its development. Current medical interventions primarily rely on steroids, but prolonged use of these medications can lead to adverse reactions, making their extended use inadvisable. Nevertheless, emerging studies suggest that probiotics have the ability to alleviate allergic symptoms by modulating the immune system [24]. In this investigation, NTU 101 was orally administered to animal subjects before inducing AD. Following the induction process, a comprehensive assessment was conducted, including the observation of skin irritation sites, histological analyses, and evaluations of the immune system. The objective was to determine the potential of NTU 101 in proactively regulating and mitigating symptoms associated with AD.

There were no noticeable differences in food intake among the various groups during the initial phase of inducing the AD model. Throughout the experimental period, the animals in the experimental group consistently gained weight. The weight changes in the NOR group and OVA group showed similar trends between the two groups. Similarly, the animals in the LGG group also exhibited steady weight gain, although slightly less than those in the NTU 101 group. In contrast, the animals in the CM group experienced a significant reduction in weight. This phenomenon can be attributed to the known side effects associated with this medication [25]. After continuous, four-week administration of the test or OVA substance to healthy animals, an investigation revealed that NTU 101 and ATCC 53103 (LGG) had no significant impact on the total IgE content present in the animal serum. However, it is important to note that prednisolone, a well-established medication, exhibited a substantial reduction in total IgE content. Changes in food intake and body weight of animals during the establishment of an AD model show correlations with the method of induction. The outcomes revealed that when animals were subjected to bandage stimulation, the body weight across all groups decreased; however, the animals resumed weight gain upon removal of the bandage. Throughout the process of inducing skin irritation, the NTU 101 group exhibited significantly higher body weight compared to the OVA group. Notably, due to the side effects associated with prednisolone usage, the group receiving CM displayed a markedly lower body weight than the OVA group. Conversely, there were no discernible disparities in body weight between the LGG group and the AD model animals. Based on the findings, it is apparent that the administration of NTU 101 during the AD model experiment played a crucial role in maintaining the animals’ body weight.

In this study, the establishment of an AD animal model was confirmed through multiple indicators. The evaluation included the AD Score (SCORE) for skin irritation site appearance, histological analysis of skin features, and the concentration of total IgE in serum [19]. During the skin irritation period, the SCORE of the OVA groups significantly exceeded that of the NOR group. The OVA group animals exhibited increased redness, swelling, damage, and excoriation during the induction period. Histopathological findings revealed heightened levels of inflammatory cell infiltration as well as increased thickness of the epidermal and dermal layers in the OVA group compared to the NOR group. Additionally, previous research demonstrated a positive correlation between the severity of AD and the presence of TSLP antigen [26]. In our study, we observed increased expression of TSLP antigen in the epidermal cell layer of the OVA group (Figure 4). Furthermore, there was a significant increase in the levels of total IgE and OVA-specific IgE in the OVA group. In summary, our findings support the conclusion that the animals induced with OVA in this experiment effectively serve as a model for AD.

In the immune response, helper T cells (Th cells) play a very important role. Under normal circumstances, the immune system in the body has a balance between Th1 and Th2 and regulates the response through regulatory T cells (Treg cells). Treg cells, formerly known as suppressor T cells, are a subpopulation of T cells that modulate the immune system, maintain tolerance to self-antigens, and prevent autoimmune disease [27]. In the immune system, the spleen is a secondary lymphoid organ that stores and filters blood and makes white blood cells that protect you from infection. In this experiment, splenocytes were taken to detect Th cell surface molecular targets and intracellular cytokines. It was found that after oral administration of NTU 101 in AD model animals, the proportion of Th2 cells in the body increased, and the Treg cell-specific transcription factor FOXP3 expressed an increase (Figure 6). FOXP3 is a marked transcription factor that plays an important role in Treg cell maturation and immunosuppressive function [28,29]. It is inferred that the test substance may regulate the allergic response caused by AD through Treg cells.

When an allergic reaction occurs, Th2 cells release IL-4, leading to an elevation in the concentration of IgE antibodies in the bloodstream. In comparison to the OVA group, there were no substantial differences in serum total IgE and OVA-specific IgE antibody levels between the NTU 101 group and the LGG group (Figure 5). However, the CM group showed a significant reduction in IgE antibody levels following the test. The results of the five leukocyte classifications indicated that there were no significant variances in the proportions of neutrophils, lymphocytes, eosinophils, and basophils between the NTU 101 group and the OVA group, while the monocyte proportion was relatively low. Because AD represents a contact allergic reaction, an array of immune responses is triggered when an allergic reaction occurs. This leads to white blood cells reaching the site of skin irritation, resulting in inflammatory cell infiltration and inflammation. Since the white blood cells in the body become activated, differences in the counts of various immune cell types in the blood cannot be observed after the test. Furthermore, there were no notable disparities in spleen weight between the NTU 101 group (0.084 ± 0.011 g) and the LGG group (0.079 ± 0.007 g) when compared to the OVA group (0.085 ± 0.009 g). However, compared to the OVA group, the CM group (0.052 ± 0.004 g) exhibited a significantly lighter and atrophied spleen (*p* < 0.001). The literature suggests that this phenomenon is associated with prednisolone medication [30].

Skin observations at the site of stimulation, AD Score (SCORE), and histopathological interpretations indicate that during the induction period, the NTU 101 group exhibited lower SCORE scores for the stimulated skin compared to the OVA group, the LGG group, and the CM group. These scores were similar to those observed in the NOR group. Histopathological results revealed that, following H&E staining of animal skin sections in the NTU 101 group, there was significantly reduced infiltration of inflammatory cells and thinner epidermal and dermal layers compared to the OVA group. Additionally, the expression of the TSLP antigen was notably lower than that in the OVA group. Notably, there was no significant difference between the LGG group and the OVA group. This suggests that the test substance aids in alleviating symptoms of AD resulting from local irritation with superior efficacy compared to the LGG group. The skin of animals in the NTU 101 group exhibited the smoothest and most complete recovery. Observation of the skin appearance of the stimulation site AD score (atopic dermatitis score, SCORE) and histopathological interpretation results showed that during the induction period, the SCORE scores of the stimulation site skin in the NTU 101 group were lower than those of the OVA group, LGG group, and CM group, and similar to the NOR group (Figure 2). Histopathological results showed that after H&E staining of animal skin sections in the NTU 101 group, the infiltration of inflammatory cells, the thickening of the epidermal cell layer, and the dermis layer were all significantly lower than those in the OVA group, and the expression of TSLP antigen was also significantly lower than that in the OVA group. There was no significant difference between the LGG group and the OVA group, so it is inferred that the test substance helps to improve the symptoms of AD caused by local irritation, and the effect is better than the LGG group. The skin of the animals in the NTU 101 group recovered the smoothest and most complete (Figure 3).

## 5. Conclusions

AD is a chronic, inflammatory skin disease. Based on the above results, it can be deduced that after oral administration of NTU 101 to normal model animals, it does not affect the autoimmune system and is conducive to the growth of animals. However, before the animals were induced into AD mode, NTU 101 was administered orally in advance, which could increase the ratio of CD4^+^/CD25^+^/FOXP3^+^ in the spleen, but the ratio of CD4^+^/IL-4^+^ also increased, while there is no difference in the ratio of CD4^+^/IFN-γ^+^ and CD4^+^/IL-17^+^, and the content of total IgE antibody in serum increases. It is deduced that NTU 101 may not regulate the balance between Th1 cells and Th2 cells but increase the FOXP3 expression of the CD4^+^/CD25^+^ ratio to promote the maturation of Treg cells, reduce the SCORE, inflammatory cell infiltration, thickening of the epidermal cell layer and dermis layer at the stimulated site, and relieve the symptoms of allergic reactions in AD.

## Figures and Tables

**Table 1:** Body weight of each group during the experimental period.

Group	Body Weight (g)											
	Day 0	Day 7	Day 14	Day 21	Day 28	Day 35	Day 42	Day 49	Day 56	Day 63	Day 70	Day 73
NOR	17.81 ± 0.61	18.68 ± 0.84	19.16 ± 0.55	19.68 ± 0.75	19.71 ± 0.65	20.04 ± 0.58	17.39 ± 0.47	19.69 ± 0.41	18.97 ± 0.97	19.47 ± 0.48	17.66 ± 0.94	19.48 ± 0.60
OVA	18.21 ± 0.50	18.44 ± 0.95	18.56 ± 1.00	19.05 ± 0.67	20.05 ± 0.66	19.63 ± 0.76	16.99 ± 0.98	19.60 ± 0.96	18.08 ± 0.84	19.61 ± 0.46	16.72 ± 0.67	19.60 ± 0.67
CM	18.35 ± 0.83	17.91 ± 0.97	17.26 ± 0.97 ^a^*	18.26 ± 1.16	18.10 ± 1.29 ^a^**	17.85 ± 0.77 ^a^***	15.86 ± 0.39 ^a^**	17.46 ± 0.57 ^a^***	16.93 ± 0.48 ^a^*	18.06 ± 1.02 ^a^***	15.76 ± 0.64	17.64 ± 0.69 ^a^***
NTU 101	18.11 ± 0.65	18.93 ± 0.52	19.10 ± 0.53	19.31 ± 0.66	19.78 ± 0.67	20.19 ± 0.70	18.19 ± 0.57 ^b^**	20.45 ± 0.37 ^b^*	19.63 ± 1.16 ^b^**	20.51 ± 0.54 ^b^*	18.06 ± 0.52 ^b^**	19.88 ± 0.59
LGG	18.24 ± 0.92	18.13 ± 0.95	18.13 ± 1.02	18.60 ± 0.94	19.36 ± 1.26	19.09 ± 0.89	16.62 ± 0.71	19.41 ± 0.76	18.49 ± 0.85	19.40 ± 0.48	17.00 ± 1.00	18.66 ± 0.75 ^c^*

Each value was expressed as the mean ± SD (*n* = 8). ^a^ is the comparison between the CM and OVA groups, ^b^ is the comparison between the NTU 101 and OVA groups, and ^c^ is the comparison between the LGG and OVA groups (*: *p* < 0.05; **: *p* < 0.01; ***: *p* < 0.001).

**Table 2:** Daily food intake of each group during the experimental period.

Group	Daily Food Intake (g)										
	Day 0	Day 7	Day 14	Day 21	Day 28	Day 35	Day 42	Day 49	Day 56	Day 63	Day 70
NOR	21.19 ± 0.54	15.55 ± 3.77	20.49 ± 2.82	17.44 ± 2.18	16.73 ± 2.09	18.31 ± 2.49	25.67 ± 3.92	23.01 ± 1.95	23.13 ± 1.70	21.42 ± 1.11	10.59 ± 0.83
OVA	20.69 ± 0.56	17.55 ± 1.02	18.21 ± 0.22	18.63 ± 0.81	15.74 ± 0.67	19.63 ± 1.18	26.43 ± 0.53	22.80 ± 0.66	23.87 ± 0.28	19.94 ± 0.96	11.82 ± 0.72
CM	20.94 ± 1.61	16.31 ± 1.49	16.72 ± 1.12	16.11 ± 0.62	15.41 ± 0.31	17.76 ± 0.64	24.11 ± 0.30	21.60 ± 0.68	22.79 ± 2.40	18.30 ± 1.16	10.88 ± 0.80
NTU 101	20.69 ± 0.43	18.53 ± 1.04	18.85 ± 0.61	18.92 ± 0.30	17.33 ± 0.04	21.60 ± 0.68	25.05 ± 0.08	22.91 ± 0.42	22.78 ± 0.04	18.68 ± 0.23	10.27 ± 0.85
LGG	19.10 ± 0.63	16.32 ± 0.61	17.45 ± 0.11	17.54 ± 0.87	14.89 ± 0.26	20.68 ± 0.33	24.94 ± 0.33	23.94 ± 0.79	21.31 ± 0.81	19.50 ± 0.15	9.80 ± 0.95

Each value was expressed as the mean ± SD (*n* = 8).

## Data Availability

All data included in this study are available upon request by contacting the corresponding authors.
